# Airborne Infrared and Visible Image Fusion Combined with Region Segmentation

**DOI:** 10.3390/s17051127

**Published:** 2017-05-15

**Authors:** Yujia Zuo, Jinghong Liu, Guanbing Bai, Xuan Wang, Mingchao Sun

**Affiliations:** 1 Changchun Institute of Optics Fine Mechanics and Physics, Chinese Academy of Science, #3888 Dongnanhu Road, Changchun 130033, China; zuoyujia@ciomp.ac.cn (Y.Z.); baigb@ciomp.ac.cn (G.B.); wangxuan@ciomp.ac.cn (X.W.); 17743017276@163.com (M.S.); 2 University of Chinese Academy of Sciences, #19 Yuquan Road, Beijing 100049, China

**Keywords:** airborne optoelectronic platform, image fusion, image segmentation, saliency extraction, dual-tree complex wavelet transform (DTCWT)

## Abstract

This paper proposes an infrared (IR) and visible image fusion method introducing region segmentation into the dual-tree complex wavelet transform (DTCWT) region. This method should effectively improve both the target indication and scene spectrum features of fusion images, and the target identification and tracking reliability of fusion system, on an airborne photoelectric platform. The method involves segmenting the region in an IR image by significance, and identifying the target region and the background region; then, fusing the low-frequency components in the DTCWT region according to the region segmentation result. For high-frequency components, the region weights need to be assigned by the information richness of region details to conduct fusion based on both weights and adaptive phases, and then introducing a shrinkage function to suppress noise; Finally, the fused low-frequency and high-frequency components are reconstructed to obtain the fusion image. The experimental results show that the proposed method can fully extract complementary information from the source images to obtain a fusion image with good target indication and rich information on scene details. They also give a fusion result superior to existing popular fusion methods, based on eithers subjective or objective evaluation. With good stability and high fusion accuracy, this method can meet the fusion requirements of IR-visible image fusion systems.

## 1. Introduction

With the development of sensor imaging technology, an unmanned aerial vehicle (UAV) carrying an infrared (IR)-visible light sensor can work in both day and night, enabling the wide use of UAVs in fields such as military reconnaissance, target localization, and forest fire control [1,2,3,4]. Infrared and visible light camera imaging mechanism differs—infrared cameras use the object thermal radiation principle imaging; visible light cameras do so according to the object of the principle of light reflection imaging. The two images are strongly complementary to each other, as an IR image has good target indication, while a visible image gives clear background information. The fusion of IR and visible images combines their respective advantages to improve UAV reliability in target detection and identification.

The current studies on IR-visible light fusion often leave the registration of source images out of consideration. However, factors such as high UAV speed, great imaging distance, and imaging sensor vibration in flight can lead to the distortion (in terms of translation, rotation, and scale) of airborne IR and visible images taken by an UAV, so as to seriously affect the fusion result. Therefore, IR and visible images must be accurately registered before being fused. Ee21existing registration methods mostly focus on algorithmic research at lower operation efficiency [5,6,7,8,9]. Registration based on the structural design and optical alignment of an airborne photoelectronic imaging system can ensure registration accuracy, and increase arithmetic speed.

By considering the image information on multiple scales, the multiresolution fusion method is effective in fusion, and has received extensive attention in the IR-visible image fusion field. Traditional multiresolution-based fusion algorithms are mainly pyramid transform and discrete wavelet transform (DWT) [10,11,12]. Strong in image reconstruction, DWT is the first to apply to the IR-visible image fusion field. However, due to translation variance and poor direction selectivity, an image fused by DWT will have insufficient details and blurry edges. In recent years, researchers have successively proposed improved algorithms and better multiresolution decomposition tools such as dual-tree complex wavelet transform (DTCWT) [13,14], Contourlet [15,16], Curvelet [17,18], and Non-subsampled Contourlet Transform, (NSCT) [19,20,21]. However, the downsampling operation used in image decomposition through Contourlet can cause the pseudo-Gibbs phenomena of a fusion image. In order to avoid this phenomenon, a non-subsampled contourlet transform (NSCT) is proposed. The fusion effect is improved, but the processing speed is too slow. In contrast, DTCWT relies on two groups of Q-shift filters, and uses two real wavelets to realize the complex wavelet transform. After being decomposed through DTCWT, an image can obtain the detail information in six directions, showing better direction selectivity than DWT. In addition, the two groups of Q-shift filters have different delays that can remove the aliasing caused by translation, so DTCWT boasts translation invariance. With limited data redundancy, DTCWT is more efficient than Contourlet.

The research on IR-visible image fusion methods focuses on keeping as much complementary information of the two images as possible. With low resolution and poor background information definition, IR images are susceptible to noise. The difficulty in IR-visible image fusion research lies in correctly extracting the information on hot targets from the IR image, and trying to keep clear background information of the visible image in the fusion image. To keep the hot-target information of the IR image, Li et al. [22] proposed a fusion method based on PCA (principal component analysis). After the image decomposition through Contourlet, low-frequency components are fused through a strategy based on PCA to keep the IR image structure, while high-frequency components are fused through the average gradient method. In the image fused by this method, the information on hot targets is relatively complete, but the information on background details is blurred. To better keep the information on background details of the visible image, Liu et al. [23] proposed the image fusion method combining PCNN (pulse-coupled neural networks) with IHS transform. In this, the image contrast was enhanced through IHS transform, low-frequency components were fused through the rule based on adaptive threshold, and high-frequency components were fused through PCNN. Although this method obtains higher contrast of the fusion image and rich information on background details, some targets of interest in the image are still not clear enough. Li et al. [24] proposed a fusion method based on guidance filter (GFF) to at first break down the source image into a basic layer and a detail layer, and then to fuse the two layers through the GFF-based weighted average method. Although these methods improve the quality of the fusion image more or less, they fail to give attention to the target indication of the IR image and the spectral features of the visible image, and leave anti-noise performance out of consideration.

This paper proposes an IR-visible image fusion method introducing region segmentation into the DTCWT region. After the quick registration of IR and visible images, segment the IR image by significance into region of interest (ROI) and background region, so that the ROI can still be correctly extracted from an IR image with low SNR. Then decompose the image into high-frequency and low-frequency components through DTCWT [13,14]. For low-frequency components, fuse them in the ROI and background region according to different fusion rules and in line with the segmentation result so that the structure of IR source image can be better kept, and the target indication of the fusion image can be highlighted. For high-frequency components, assign the weight for every region by the richness of detail information, fuse high-frequency components through a 2D edge matrix based on region weights and segmentation results, and then differentiate meaningful detail information from noise through a shrinkage function to remove noise. In this way, the detail information in the visible source image can be better kept, and noise effectively suppressed. Finally, obtain the fusion image through DTCWT inverse transform.

## 2. Fusion System on the Airborne Photoelectric Platform

**Figure 1:**
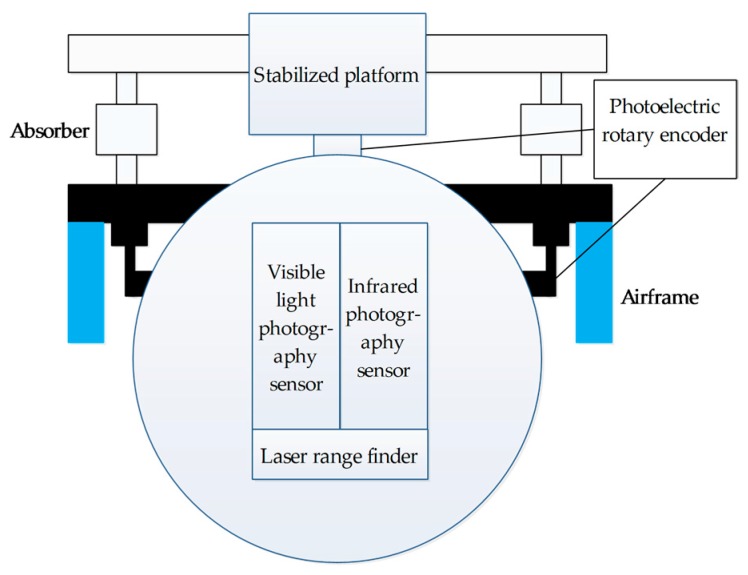
Structure of the airborne photoelectric platform.

The airborne photoelectric platform is a kind of photoelectric reconnaissance equipment loaded on UAVs and other aircraft. As shown in the Figure 1, the airborne photoelectric platform fusion system adopted by this paper mainly consists of a visible light camera, an infrared thermal imager (IF), a laser range finder (LRF), a gyrostabilized platform, a image processing module, a recorder, and a wireless link. Visible light camera and infrared thermal imager are used to obtain images. The LRF can measure the distance between the airborne photoelectric platform and the ground target, which is used to locate the ground target. The gyroscope controls the steering of the camera through the cooperation of the servo control system and the image processing module to isolate the disturbance of the UAV, so that the images obtained by the infrared camera and the visible light camera mounted on the UAV are clear and stable. The recorder is used to record the original image video taken by the infrared camera and the visible light camera. The recorded original image will be used as a reference for the subsequent fusion accuracy if it is not high enough, the fusion effect is not good enough, or the visible and infrared original image target characteristics are highlighted without the need for complementary information fusion. The airborne photoelectric platform is connected with the ground station through the wireless link. The platform accepts the ground station control command via the wireless link, and transfers the image and platform control parameters to the ground station.

Airborne photoelectric platform is used to obtain images, and the ground station to observe the visible or infrared images, with digital or manual control search found that the target. After detecting the target via the platform, lock it to the FOV (field of view) center, control the camera direction through the coordination of servocontrol system and image processing module to keep the target at the FOV center, then use image processing module through the GPU for image registration and fusion processing, and then observe, track and image the target.

It is hard to avoid the vibration of airborne photoelectric platform in the imaging process as a result of UAV flight. The platform vibration can lead to the distortion (in terms of translation, angle and scale) of the images captured by IR and visible light sensors. Therefore, the images shall be accurately registered before being fused. The operating range of airborne photoelectric image fusion systems is usually at least several thousand meters. As the result of harsh shooting conditions, the imaging quality is affected by airframe vibration, atmospheric disturbance, defocus blur, haze, and other factors, so that the obtained aviation images are seriously blurred with bad contrast. On the other hand, the frequent use of this system to identify and track the targets requires superior target indication of fusion images, which raises a higher requirement for image fusion. By considering the image information on multiple scales, the multiresolution-based fusion method locks richer information in the fusion images and thus is applicable to aviation image fusion. To obtain the fusion images with highlighted targets and clear-cut scene environment, this fusion method needs strategy improvement and anti-noise consideration. Therefore, this paper proposes a fusion strategy based on ROI in the DTCWT region, which highlights the target indication of IR images in low-frequency components and detail information of visible images in high-frequency components. The method also fully considers the anti-noise performance to effectively suppress the influence of noise on the quality of fusion images.

## 3. Fast Registration of IR and Visible Images

The airborne photoelectric platform is always moving in the imaging process. The vibration and angular variation caused by flight, and the scale variation resulting from the error of focusing, renders the captured IR and visible images out of full alignment. Thus, registration shall precede the fusion. As the IR and visible images captured by airborne photoelectric platform are distorted (in terms of translation, angle, and scale), the registration algorithm shall be provided with rotation invariance and scale invariance. Moreover, it shall be efficient in operation to meet the engineering demand.

In this paper, optical alignment is used to register IR and visible images. As the resolution of a visible image is often higher than that of an IR image, the former shall be lowered to the same as the latter. Further, the design of IR and visible images share the same optical axis and path, and it is used to ensure a common LOS between optical paths. However, in the processes of manufacture, adjustment, and installation, some deviation in sensor installation location will render 2 FOV centers completely out of alignment. To solve this problem, the star method is used to mark and align the FOV centers of the two images to obtain their direct affine transformation matrix quickly, thus realizing the fast registration of IR and visible images.

**Figure 2:**
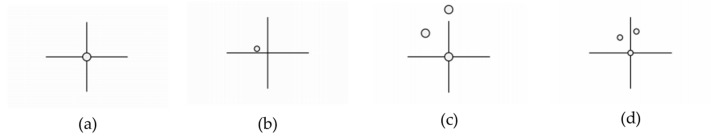
Principle of star method. (**a**) Visible star point; (**b**) Infrared star point; (**c**) Visible star points; (**d**) Infrared star points.

The principle of the star method is shown in Figure 2, where the star in the visible light FOV is illustrated in Figure 2a,c, while the star in the IR FOV is illustrated in Figure 2b,d.

**Figure 3:**
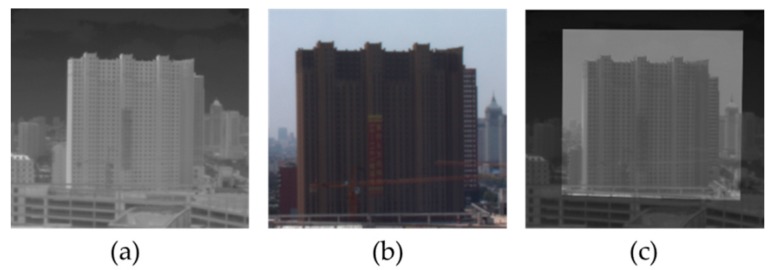
Registration effect. (**a**) Infrared (IR) image; (**b**) Visible image; (**c**) Registered image.

When a star is observed through both visible light and IR light, it is found at the center of visible light FOV, but somewhat off the IR FOV center. The translation between the two images can be derived from this deviation. Although, when the star is turned to 3 different positions, the triangle formed by the 3 star points observed in the visible light FOV will deviate from that in the IR FOV. In this case, the degrees of rotation and zooming between the two images can be determined through the direct relationship between the two triangles. The registration effect is shown in Figure 3.

This registration method can register the two images as soon as the data are read out of the sensors, with time taking only 2 ms. With simple operation, efficient computation, high registration accuracy (the registration precision can reach the pixel level), and good stability (only the error matching frame will be changed when the zoom lens group is switched), it can satisfy the registration demand of the image fusion system.

## 4. IR-Visible Image Fusion Method Combined with Region Segmentation

**Figure 4:**
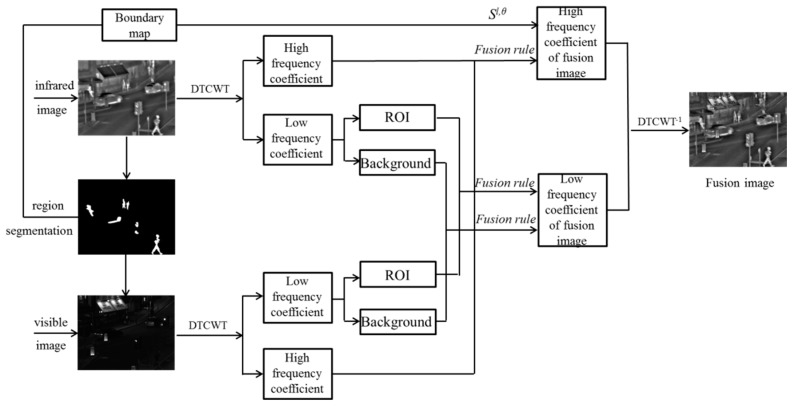
Fusion algorithm flowchart.

The flow chart of this algorithm is shown in Figure 4. Firstly, segment the region in the IR image by significance to obtain its ROI and background region. Then map the segmentation result to the visible light image. Transform IR and visible images through DTCWT. Every transform can produce two low-frequency coefficients {*L^l^*^,1^, *L^l^*^,2^} and the high-frequency coefficients in six directions {*H^l,θ^*, *θ* = ±15°, ±45°, ±75°}, where *l* is the number of DTCWT decomposition layers. Low-frequency coefficients reflect the essential information of an image, while high-frequency coefficients show its detail information. Low-frequency components are fused in the ROI and background region under different rules, according to the image segmentation result. High-frequency components are fused under a rule that considers the weight assigned for every region by the richness of detail information. Section 4.1 of this paper introduces the region segmentation method based on ROI, while Section 4.2 and Section 4.3 describe low-frequency and high-frequency fusion rules, respectively.

### 4.1. Image Region Segmentation Based on ROI

**Figure 5:**
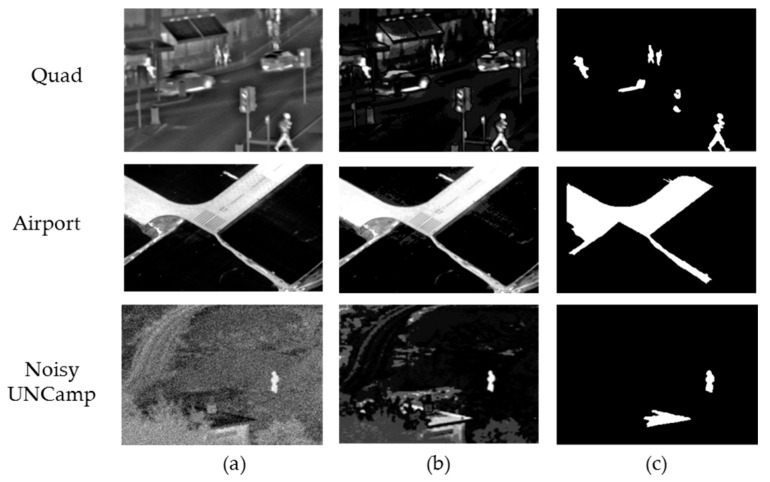
Region segmentation result based on region of interest (ROI): (**a**) infrared (IR) image; (**b**) Significance map of the IR image; (**c**) Region segmentation result based on ROI.

Because the IR image provides the information on object heat radiation connected with good target indication, this paper segments the region in the IR image at first to obtain the ROI and background region, and then fuses IR and visible images according to the region features. In recent years, image segmentation has also had a great amount of research focus in the image processing field. Methods such as region growing [25,26] and K-means [27,28] are commonly used in the IR image segmentation, but are not very effective for the aviation IR images with low SNR and complex background information. This paper proposes a region segmentation strategy based on ROI that will at first extract significant features from the IR image, and then segment the image according to the significance result. After the significance extraction, the information on a hot target will be enhanced and the background information will be blurred, allowing a stronger contrast for an entire IR image. This strategy can effectively suppress the influence of noise on IR image segmentation. Cheng et al. [29] proposed a ROI detection method based on global contrast. Aiming to separate a large target area from its surroundings, their method is superior to local contrast methods showing higher significance only near the contour and that processes the images very quickly. By using the color contrast between image pixels to define the significance value, the pixel-significance detection method based on histogram contrast (HC) was proposed. The significance of pixel I*_c_* in the image I is defined as:

(1) $$S_{hc}(I_{c})=\sum_{\forall I_{i}\in I}Dis(I_{c},I_{i})$$

where $Dis(I_{c},I_{i})=\|I_{c}\;-{\;I}_{i}\|$ is the color distance of I_c_ in the Lab space, indicating the color difference between I_c_ and I_i_. Then, the Equation (1) can be rewritten as:

(2) $$S_{hc}(I_{c})=S_{hc}(a_{c})=\sum_{j=1}^{n}f_{j}Dis(a_{c},a_{j})$$

where a_c_ is the color value of pixel I_c_, n is the total number of colors contained in the image, and *f_j_* is the occurrence probability of a_j_ in the image. This paper applies the above method to the significance extraction and segmentation of an IR image. In doing so, the Equation (2) is changed to calculate the significance of every gray level. The extraction of significance from an IR image is mainly to highlight the information on a hot target and to clearly distinguish it from background information. To increase the calculation speed, this paper proposes to change the gray values 0–255 to 0–15. In the Equation (2), there will be *n* = 16, showing that the gray difference of 16 pixels/scale can meet the requirement of hot-target significance detection in the IR image. The significance map quantified in this way may have some blemishes in the form of gray value jumping, but this can be improved through smoothening. Convert the significance value of every gray level into the weighted average significance value of similar gray levels. Choose the significance values of 4 adjacent gray levels to improve the significance of pixel a through the Equation (3):

(3) $${S_{hc}}^{\prime }(a)=\frac{1}{3L}\sum_{i=1}^{4}\left(L-Dis(a,a_{i})\right)S(a_{i})$$

where $L=\sum_{i=1}^{4}Dis(a,a_{i})$is the distance between gray level a and its four nearest gray levels a_i_. Thus, in the gray space, a bigger weight will be assigned to the gray level closer to a, and similar gray levels will be assigned to similar significance values. In this way, the flaws caused by quantification can be reduced. After obtaining the ROI through significance detection, we shall segment the region in the IR image with the help of ROI, take the initial segmentation result obtained after the binarization of significance map as the initial image, and conduct iterative operation through GrabCut [30] to further optimize the segmentation result. Every iterative operation is followed by corrosion and dilation operation to obtain a new target region, which, in turn, is followed by the next iteration. In this paper, no more than four iterations are allowed. The ROI-based region segmentation result in three different situations is shown in Figure 5. Quad is the infrared image of multiple targets in the urban environment, Airport is the real aerial image, and Noisy UNCamp is a complex field background infrared image and artificially added Gaussian noise.

In Figure 5, it can be clearly seen that in the segmentation result, the ROI of the IR image has been completely extracted, and it can obtain the correct segmentation result for the complex background and the low SNR (signal to noise ratio) image. Thus, this lays a foundation for subsequent fusion to highlight the target direction of the IR image. Table 1 shows the calculation time of the three sets of image segmentation methods in Figure 5. The experiment was compeleted in the Microsoft Visual 2010 environment, where the computer CPU is Intel core i3-2120, 3.3 GHz, 4 G.

### 4.2. Low-Frequency Fusion Rule

After the multiresolution decomposition, low-frequency components of an image will reflect the essential information of the image, while high-frequency components will show its detail information. Most of the IR-visible image fusion methods based on multiresolution decomposition focus on how to design the fusion rule of high-frequency components. Low-frequency components are mainly fused through simple weighted averaging or arithmetic averaging. For aviation IR-visible image fusion, it is important to acquire the complementary information of the two images, namely, the information on a hot target in the IR image and on clear edges, background, and other details of the visible image. Therefore, the ability to acquire complementary information for the fusion image depends more on the fusion effect of low-frequency information. The majority of existing low-frequency fusion rules are based on simple weighted averaging and arithmetic averaging, which will bring down the contrast of IR hot-target information in the fusion image. If the target brightness is enhanced by increasing the low-frequency coefficient weight of the IR image through weight assignment, part of the detail information in the visible image will be lost. To better preserve the information on a high-brightness target in the IR image and the information on clear background details of the visible image, this paper proposes a new low-frequency fusion rule based on ROI region segmentation. At first, segment the IR image into a ROI and a background region by using the image segmentation method, as described in Section 4.1. Then map the segmentation result to the visible light image, and design different fusion rules to fuse low-frequency components in the ROI and background region of the two images.

To achieve the goal of low-frequency component fusion, we shall keep as much information as possible on hot-target brightness of the IR image in the ROI, and as much information as possible on details and scene of the visible image in the background region. Therefore, the low-frequency coefficient of the IR image is directly chosen as the low-frequency fusion coefficient in the ROI, as expressed by the Equation (4):

(4) $$L_{F}^{l,i}(x,y)=L_{ir}^{l,i}(x,y)\;\;(x,y)\in ROI$$

where $L_{F}^{l,i}$ is the low-frequency coefficient of the fusion image (*i* = 1, 2). $L_{ir}^{l,i}$ is the low-frequency coefficient of the IR image at the layer *l*.

For the background region, an adaptive weighted fusion rule based on region variance is proposed. Region variance shows how fast a pixel in the region changes. A bigger region variance means a greater change of the pixel, higher contrast, and richer information in the region. Therefore, pixels with a bigger region variance shall have a greater weight in the fusion image. According to the above principle, an image fusion rule based on the adaptive weighting of region variance can be established:

(5) $$L_{F}^{l,i}(x,y)=\omega_{ir}L_{ir}^{l,i}(x,y)+\omega_{vis}L_{vis}^{l,i}(x,y)$$

where $L_{F}^{l,i}$ is the low-frequency coefficient of the fusion image (*i* = 1, 2). $L_{ir}^{l,i}$ is the low-frequency coefficient of the IR image at the layer *l*. $L_{vis}^{l,i}$ is the low-frequency coefficient of the visible image at the layer *l*. The weight ω is determined by both region variance σ and correlation coefficient *r*, whose definitions are shown in Equations (6) and (7) respectively:

(6) $$\sigma^{2}=\frac{1}{M\times N}\sum_{i=1}^{M}\sum_{j=1}^{N}{\left(I(i,j)-\bar{x}\right)}^{2}$$

(7) $$r=\sum_{i=1}^{M}\sum_{j=1}^{N}\frac{\left(I_{ir}(i,j)-{\bar{x}}_{ir})\times (I_{vis}(i,j)-{\bar{x}}_{vis}\right)}{\sqrt{\left(\sum_{i=1}^{M}\sum_{j=1}^{N}{\left(I_{ir}(i,j)-{\bar{x}}_{ir}\right)}^{2})\times (\sum_{i=1}^{M}\sum_{j=1}^{N}{\left(I_{vis}(i,j)-{\bar{x}}_{vis}\right)}^{2}\right)}}$$

where $\omega_{vis}$ is the weight of low-frequency coefficient of the visible image; $\omega_{ir}$ is the weight of low-frequency coefficient of the IR image; σ is region variance in a window whose size is chosen by this paper as 3 × 3; *r* is correlation coefficient; I_ir_ and I_vis_ are IR image and visible image, respectively, in a size of M × N; $\bar{x}$ is average gray level of the image. The weights in Equation (5) are assigned as below:

(8) $$\omega_{vis}=\left\{ \begin{array}{l} 0.5,\\ 1-r\\ r, \end{array}\right.,\begin{array}{l} r\geq 0.5\\ r<0.5\;\&\;\sigma_{\mathrm{vis}}^{2}\geq \sigma_{\mathrm{ir}}^{2},\;\\ r<0.5\;\&\;\sigma_{\mathrm{vis}}^{2}<\sigma_{\mathrm{ir}}^{2} \end{array}\omega_{ir}=1-\omega_{vis}$$

where $\omega_{vis}$ is the weight of low-frequency coefficient of the visible image, $\omega_{ir}$ is the weight of low-frequency coefficient of the IR image, σ is region variance, and *r* is correlation coefficient.

### 4.3. High-Frequency Fusion Rule

After the multiresolution decomposition, high-frequency components will reflect the information on edge, contour, and other details of the decomposed image. To retain as much information on details and texture of the source image as possible, this paper proposes a new high-frequency fusion rule based on region weighting. Through the above image segmentation method based on ROI, the IR and visible images are segmented into n regions, namely, R = {r_1_, r_2_,…,r_n_}. The region weight *Q* is defined as:

(9) $$Q=\left\{Q_{r_{1}},Q_{r_{2}},\cdot \cdot \cdot Q_{r_{n}}\right\}$$

The region weight *Q* corresponds to every small region r*_i_* (*i* = 1, 2…*n*), and is calculated through the following equation:

(10) $$Q(r_{i}^{\theta })=\frac{1}{\left|r_{i}^{\theta }\right|}\sum_{\forall l,(x,y)\in r_{i}^{\theta }}\left|H^{l,\theta }(x,y)\right|$$

where $H^{l,\theta }(x,y)$ is the high-frequency coefficient derived from post-DTCWT image decomposition, *l* is the number of decomposition levels (*l* = 1, 2, 3, 4), θ is the decomposed directional sub-band (*θ* = ±15°, ±45°, ±75°), and $\left|r_{i}^{\theta }\right|$ is the absolute value of region $r_{i}^{\theta }$. The fusion rule *f* is defined according to the *Q* value calculated through the Equation (10). According to the fusion rule *f*, to ensure richer texture information in every subregion of the fusion image, the region with a bigger *Q*_r*i*_ in IR and visible images is chosen to calculate the fusion coefficient of region r*_i_* in the fusion image, and the average of high-frequency coefficients in the former region is taken as the high-frequency coefficient of region r_i_. To ensure that the edge details of every subregion in the fusion image are more remarkable and clearer, the binary edge matrix $S^{l,\theta }$ is introduced as weight into the calculation of high-frequency coefficient. To ensure the edge continuity and integrity of every subregion, $S^{l,\theta }$ shall be obtained through the dilation operation and 2D average filtering of the ROI-based image segmentation result, described in Section 2 of this paper. The high-frequency coefficient of the fusion image is rewritten as:

(11) $$H_{F}^{l,\theta }=(1-S^{l,\theta })\sum_{R}f(H_{ir}^{l,\theta },H_{vis}^{l,\theta })+S^{l,\theta }\max(H_{ir}^{l,\theta },H_{vis}^{l,\theta })$$

In the image fusion process, the edge ups and downs may lead to distortion of the fusion image. The phase of DTCWT transform coefficient corresponds to the exact location of its directional feature in the region. Therefore, to reduce the distortion caused by edge ups and downs, the high-frequency coefficient derived from IR-visible image DTWCT is used to calculate a unit vector and the expected high-frequency coefficient of the fusion image with improved average phase, as shown in the following rewritten form:

(12) $${\tilde{H}}_{F}^{l,\theta }(x,y)=\frac{H_{ir}^{l,\theta }(x,y)+H_{vis}^{l,\theta }(x,y)}{\left|H_{ir}^{l,\theta }(x,y)+H_{vis}^{l,\theta }(x,y)\right|}\left|H_{F}^{l,\theta }(x,y)\right|$$

The environment where the aviation images are taken is very complex, so the high-frequency information of these images often contains some noise. This paper uses the shrinkage function proposed by Loza et al, through derivation, as the maximum posteriori estimation [31] to reduce the influence of noise on high-frequency information. Suppose the shrinkage function is T*_s_*. To highlight the contrast of image details, a given value C (C > 1) is set to increase the high-frequency coefficient. But this simple method will amplify the remaining noise as well. To tackle this problem, we shall only increase the high-frequency coefficient for those pixel points capable of being connected into a large region, while defining a binary matrix $M^{l,\theta }$. When $\left|H_{F}^{l,\theta }\right|>\tau$, there will be $M^{l,\theta }$ = 1. Then the isolated points whose value is 1 are removed from the matrix $M^{l,\theta }$. Finally, the high-frequency coefficient of the fusion image becomes:

(13) $$H_{F}^{l,\theta }=(C^{l,\theta }M^{l,\theta }+(1-M^{l,\theta }))T_{s}^{l,\theta }{\tilde{H}}_{F}^{l,\theta }$$

The low-frequency and high-frequency coefficients determined through the above fusion rule undergo the DTCWT inverse transform to finally obtain the fusion image.

## 5. Analysis of Experimental Results

To verify the correctness and effectiveness of above theoretical analysis and of the proposed method, this paper tests a large number of IR and visible images. For lack of space, only three groups of representative images are listed here for experimental verification. The first group of tested images is the “Quad” in the IR-visible image fusion dataset, the second group is real aerial image named “Airport”, and the third group is the “UNcamp” in the image fusion dataset, to which white Gaussian noise has been added. (Source of IR and visible image dataset: http://www.imagefusion.org/) The experiment was done in the Matlab 2014a environment, where the computer CPU is Intel core i3-2120, 3.3 GHZ, 4 G.

To further demonstrate the performance of the proposed method, this paper chooses some typical image fusion methods used in recent years for comparison. The comparison methods include: classic multiresolution decomposition image fusion method DWT [12]; the PCA-MST method proposed by Li et al. [22], which bases image multiresolution decomposition on Contourlet transform domain and determines the low-frequency fusion coefficient through PCA and the high-frequency fusion coefficient through the average gradient method; the HIS-PCNN method proposed by Liu et al. [23], which enhances the image contrast through IHS transform and fuses low-frequency components through the adaptive threshold rule and high-frequency components through PCNN; and the GFF method proposed by Li et al. [24], which breaks down the source image into a basic layer and a detail layer and then fuses the two layers by using the GFF-based weighted average method.

### 5.1. Evaluation Indicators

To fully demonstrate the computational efficiency and the fusion accuracy of our method, we objectively evaluate the fusion effect of our method and other comparative methods by using running time and image standard deviation (SD), information entropy (IE), average gradient (AG) and edge-preserving degree (Q^AB/F^) separately. SD is the measure of dispersion of relative gray values. The bigger the SD, the better the image contrast and the higher the fusion quality. IE is the measure of amount of image information. The bigger the IE, the richer the information contained in the fusion image. AG is the measure of expressive ability of minor image details. The bigger the AG, the stronger their expressive ability. Q^AB/F^ is the measure of how well the fusion image preserves the information on edge details of the source image. The bigger the Q^AB/F^, the higher the fusion quality.

### 5.2. Experiment of Image Fusion Contrast

#### 5.2.1. Experiment 1: Quad Image Fusion Comparison

**Figure 6:**
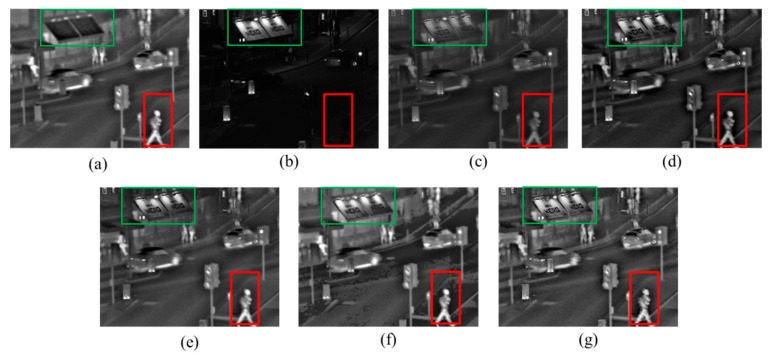
Quad image fusion comparisons: (**a**) IR image; (**b**) Visible image; (**c**) discrete wavelet transform (DWT) [12]; (**d**) PCA-MST [22]; (**e**) guidance filter (GFF) [24]; (**f**) IHS-PCNN [23]; (**g**) The proposed method.

**Figure 7:**
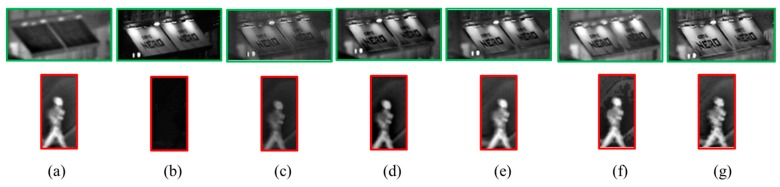
Fusion detail comparisons: (**a**) IR image; (**b**) Visible image; (**c**) DWT [12]; (**d**) PCA-MST [22]; (**e**) GFF [24]; (**f**) IHS-PCNN [23]; (**g**) The proposed method.

This paper at first chooses images from the representative Quad dataset to verify the effectiveness and fusion effect of the proposed method. Size of Quad is 640 × 496. Quad infrared and visible images are shown in the Figure 6a,b respectively. In the IR image, pedestrians, signal lights and running cars can be seen clearly, while other background information is quite blurred. In contrast, in the visible light image for the same scene, the pedestrians in the dark can't be seen clearly, but the billboard information completely invisible in the IR image is legible here. Then the UNcamp images are fused by using the proposed method and other four methods including DWT [12], PCA-MST [22], IHS-PCNN [23] and GFF [24]. The results of fusion comparisons are shown in the Figure 6. The fusion details of important complementary information (billboards and pedestrians) in the IR and visible images are compared in the Figure 7.

It can be subjectively seen from Figure 6 and Figure 7 that the five fusion methods can keep most of the important complementary information of source images, and fuse the source image information very well. However, the fusion image obtained through DWT has a blurred target edge and low image contrast, and loses much background detail information of the visible-light source image. The PCA-MST method can keep all of the target information of the IR image, but can’t capture all the scene information of the visible image, so that the billboard words are very blurred. The GFF and IHS-PCNN methods, as a whole, can keep all the target information of the IR image and the background information of the visible image. However, the fusion image obtained through HIS-PCNN shows obvious zoning and partial distortion, and the fusion image formed through GFF has insufficient scene detail information and bad contrast, compared with the method proposed by this paper. Taken together, with high image contrast, well-preserved target brightness, high background contrast, and rich detail information, the fusion image obtained through the proposed method has captured target information of the IR image and background information of the visible image. Thus, it has achieved a good visual effect.

Table 2 evaluates the performance of various fusion methods illustrated in Figure 6, by using the computation time and four objective evaluation indicators: standard deviation (SD), information entropy (IE), average gradient (AG), and edge-preserving degree (Q^AB/F^). Judging from the evaluation indicators in Table 1, all the indicators of the fusion image obtained through the proposed method are higher than those of the other 4 methods, except for SD, which is slightly lower than that of IHS-PCNN. This is mainly because the fusion image obtained through IHS-PCNN is partly distorted, leading to contrast change in the distorted region, and an increase in image SD. Therefore, the evaluation result of the proposed method is the best in general.

#### 5.2.2. Experiment 2: Aviation IR-Visible Image Fusion Comparison

**Figure 8:**
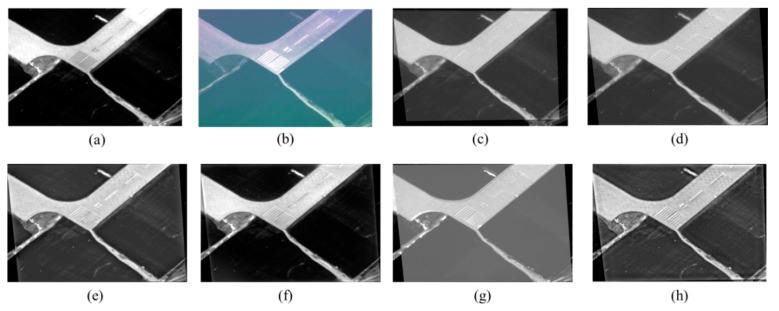
Airport image fusion comparisons: (**a**) IR image; (**b**) Visible image; (**c**) Registered image; (**d**) DWT [12]; (**e**) PCA-MST [22]; (**f**) GFF [24]; (**g**) IHS-PCNN [23]; (**h**) The proposed method.

Since the proposed method is expected to fuse aviation images taken by IR and visible light sensors on airborne photoelectric platforms, a set of IR and visible-light runway images (named “Airport“ in this paper, with the Size of Airport being 640 × 436) taken by an UAV photoelectric platform was chosen for Experiment 2. The infrared image was taken by a long-wave infrared sensor. The pixel size was 15 μm, the resolution 640 × 512, and the focal length *f* = 235 mm. The visible image was taken by the visible light sensor. The pixel size was 4.65 μm, the resolution was 1024 × 768, the focal length *f* = 72.9 mm, the experimental weather conditions were clear, and the flight height AGL was 2.3 km. Vibration and flying angle change in UAV flight can cause translation, angular rotation, scale zooming, and other forms of distortion of images captured by IR and visible light sensors, so that a whole fusion method may fail. Thus, these images must be registered before being fused. This paper registered the IR and visible images through the method described in Section 3, and then fused them for experimental purposes. The IR and visible images taken by the photoelectric platform are shown in Figure 8a,b, respectively. It is observed that the runway in the IR image is well-defined, and the marking information on the runway is very clear in the visible image. The Airport images were fused through the proposed method and other four methods, including DWT [3], PCA-MST [4], IHS-PCNN [8], and GFF [9], separately. The results of fusion comparisons are shown in Figure 8.

It can be clearly seen from Figure 8 that, in the fusion images obtained through DWT and PCA-MST, ghosting and distortion occurs in some areas, and most of the visible-image information is lost. In the fusion image obtained through IHS-PCNN, almost all of the detail information of visible-light source image is lost, the marks on the runway are blurred, and obvious zoning is seen. Only the GFF and the proposed method have a good fusion effect, but the GFF provides unclear information on runway marking details in the visible image. Taken together, in this experiment, the method proposed by this paper has the best fusion effect. Table 3 gives the computation time and fusion evaluation results of the proposed method and four other comparative methods. It can also be clearly seen from Table 3 that the proposed method behaves best in four objective evaluation indicators, showing excellent fusion performance.

#### 5.2.3. Experiment 3: Anti-Noise Performance Experiment

**Figure 9:**
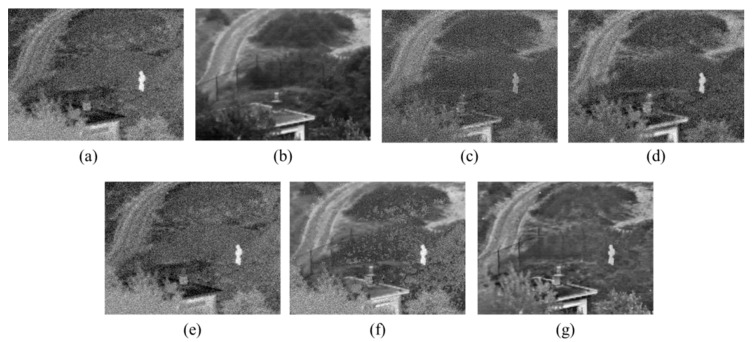
Noisy UNCamp noise-adding image fusion comparisons: (**a**) IR image; (**b**) Visible image; (**c**) DWT [12]; (**d**) PCA-MST [22]; (**e**) GFF [24]; (**f**) IHS-PCNN [23]; (**g**) The proposed method.

As a UAV flies under volatile conditions and the information on air-to-ground target background is complex, the images captured by the airborne photoelectric platform often contain noise. To demonstrate the anti-noise performance of the proposed method, white Gaussian noise is added to a group of IR and visible images belonging to the fusion datasets UNCamp, as shown in Figure 9a. The noise-adding image is named Noisy UNCamp in this article, and the size of the image is 360 × 270. The noise-adding image fusion results of the proposed method and four other comparative methods are compared in Figure 9.

It is observed from Figure 9 that the proposed method has the best anti-noise performance, while the other fusion methods are affected by noise, so that their fusion quality is generally lower. Given a low SNR of tested images, the proposed method can still keep all the target region information of source images and richer background information, providing the fusion image with the best visual effect. In Table 4, the fusion performance of the proposed method and four other comparative methods is evaluated through four objective evaluation indicators. As shown in Table 4, all the indicators of the proposed method are higher than those of the comparative methods, except for IE and AG, which are slightly lower than those of comparative algorithms. This, however, is because IE is the measure of image information richness, and AG is the measure of expressive ability of image details. The comparative algorithms are susceptible to noise, so their fusion images contain more noise, which increases the information on image details and, accordingly, forces the relevant evaluation indicators up. This means the anti-noise performance of comparative algorithms is lower than that of the proposed algorithm, whose fusion performance further proves to be excellent.

## 6. Conclusions

To solve the problems that IR-visible fusion images on airborne photoelectric platforms are faced with (such as bad contrast, blurred background details, and susceptibility to noise), this paper proposes a new fusion method combined with region segmentation. In terms of low-frequency components, this method is combined with the significance-guided region segmentation to set up a fusion rule that can highlight the hot-target information of IR images, and keep the background detail information of the visible image. In terms of high-frequency components, this paper proposes a fusion rule based on region weighting. This rule strengthens the weights of those regions with rich detail information in the fusion image according to the self-defined region weights, uses adaptive phases to correct the fusion image distortion induced by edge deformation, effectively retains the detail information of source images, and introduces a shrinkage function to enhance the anti-noise performance of this fusion method. The experimental result shows that the proposed method has efficiently extracted complementary information from IR and visible images, to generate a fusion image with a clear target, moderate brightness, clear background details, and effective noise suppression. According to the evaluation data, the proposed method has a better fusion effect and somewhat higher fusion accuracy compared with several representative fusion methods used in recent years. With a high algorithm stability, the proposed method can be applied to diversified scenes to meet the operational requirements of fusion system on an airborne photoelectric platform. Thus, it has a high application value.

## Figures and Tables

**Table 1:** The Calculation time of region segmentation method based on ROI.

Image	Quad	Airport	Noisy UNCamp
Resolution	640 × 496	640 × 436	360 × 270
Time/s	0.036	0.029	0.019

**Table 2:** Evaluation of Quad IR-visible image fusion performance.

Method	SD	IE	Q^AB/F^	AG	Time/s
DWT [12]	21.4123	5.8733	0.2108	2.6492	1.943
PCA-MST [22]	31.2098	6.3987	0.2974	3.7382	1.019
GFF [24]	32.0211	6.4520	0.2752	3.2129	1.373
IHS-PCNN [23]	**39.5745**	6.6929	0.3269	3.6875	4.693
Proposed	**35.0284**	**6.8894**	**0.5645**	**5.3487**	**0.977**

Note: Bold values are used to show the best quality of objective criterion.

**Table 3:** Evaluation of aviation IR-visible image fusion performance.

Method	SD	IE	Q^AB/F^	AG	Time/s
DWT [12]	40.3748	5.1037	0.2964	3.0552	1.526
PCA-MST [22]	48.6327	6.3254	0.3978	3.7553	**0.847**
GFF [24]	49.2637	7.0035	0.6755	3.3247	0.916
IHS-PCNN [23]	45.9653	6.1276	0.4458	3.7514	3.932
Proposed	**53.3248**	**6.9054**	**0.6326**	**6.3072**	**0.852**

Note: Bold values are used to show the best quality of objective criterion.

**Table 4:** Evaluation of Field (noise-adding) IR-visible image fusion performance.

Method	SD	IE	Q^AB/F^	AG	Time/s
DWT [12]	33.6973	7.0965	0.3575	16.4053	0.960
PCA-MST [22]	37.3488	7.2314	0.3204	**16.6699**	**0.499**
GFF [24]	37.9777	**7.2664**	0.3870	16.6331	0.693
IHS-PCNN [23]	37.2536	7.1973	0.4544	11.2374	3.137
Proposed	**39.3742**	7.2013	**0.6447**	15.2238	**0.547**

Note: Bold values are used to show the best quality of objective criterion.
